# Supplementary material for: Decreased ENSO post-2100 in response to formation of a permanent El Niño-like state under greenhouse warming
Source: Nat Commun. 2024 Jul 10;15:5810. doi: 10.1038/s41467-024-50156-9 (PMC11237138; doi:10.1038/s41467-024-50156-9)
Supplement: Supplementary file 1 — Supplementary Information [file 41467_2024_50156_MOESM1_ESM.pdf]

Supplementary Information for

A transition to a permanent El Niño-like state under persistent global warming

Tao Geng[1], Wenju Cai[2,1,3,4*], Fan Jia[5*], and Lixin Wu[1,2].

[1]Laoshan Laboratory, Qingdao, China.

[2]Frontiers Science Center for Deep Ocean Multispheres and Earth System (FDOMES) and Key Laboratory of Physical Oceanography, Ocean University of China, Qingdao, China.

[3]CSIRO Environment, Hobart, Australia.

[4]State Key Laboratory of Loess and Quaternary Geology, Institute of Earth Environment, Chinese Academy of Sciences, Xi'an, China.

[5]CAS Key Laboratory of Ocean Circulation and Waves, Institute of Oceanology, Chinese Academy of Sciences, Qingdao, China.

*Correspondence to: Wenju Cai (wenju.cai@csiro.au) and Fan Jia (jiafan@qdio.ac.cn).

This PDF file includes:

Supplementary Figs. 1 to 11

Supplementary Table 1

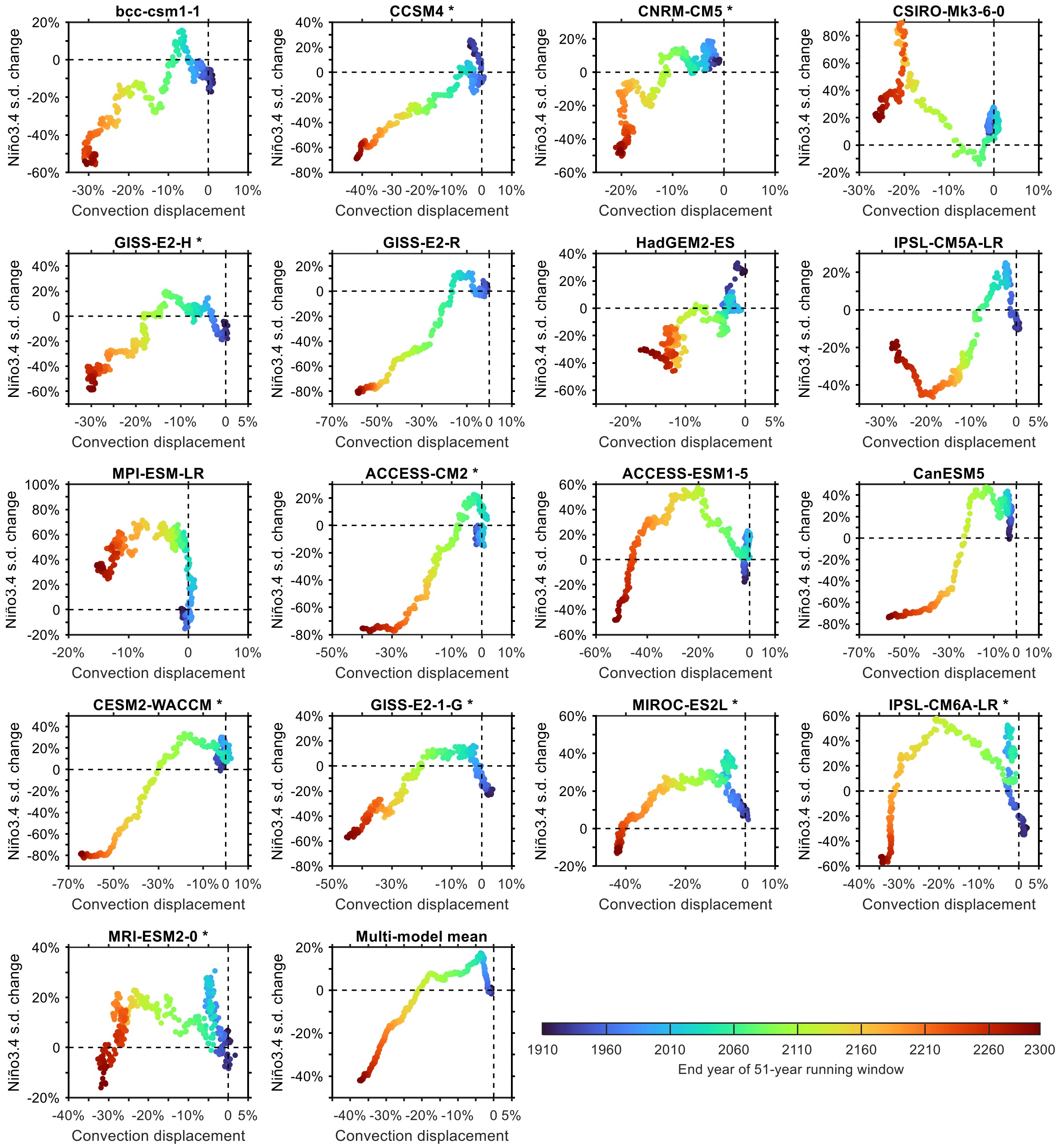

Supplementary Fig. 1 | Evolution of ENSO variability in individual models. Shown are 51-year running standard deviations (s.d.) of DJF Niño3.4 SST as a function of mean ONDJF latitudinal position of the ITCZ and the SPCZ, both referenced to a piControl 100-year rolling mean and expressed in percentage, for each individual model under RCP85/SSP585. Negative values of the convection centres displacement indicate an equatorward movement of the Pacific convergence zones. Color denotes the end year of the 51-year running window. In majority of

models, ENSO variability reverses from an initial increase to a subsequent decrease with the equatorward movement of the Pacific convergence zones, although the timing of reversal differs.

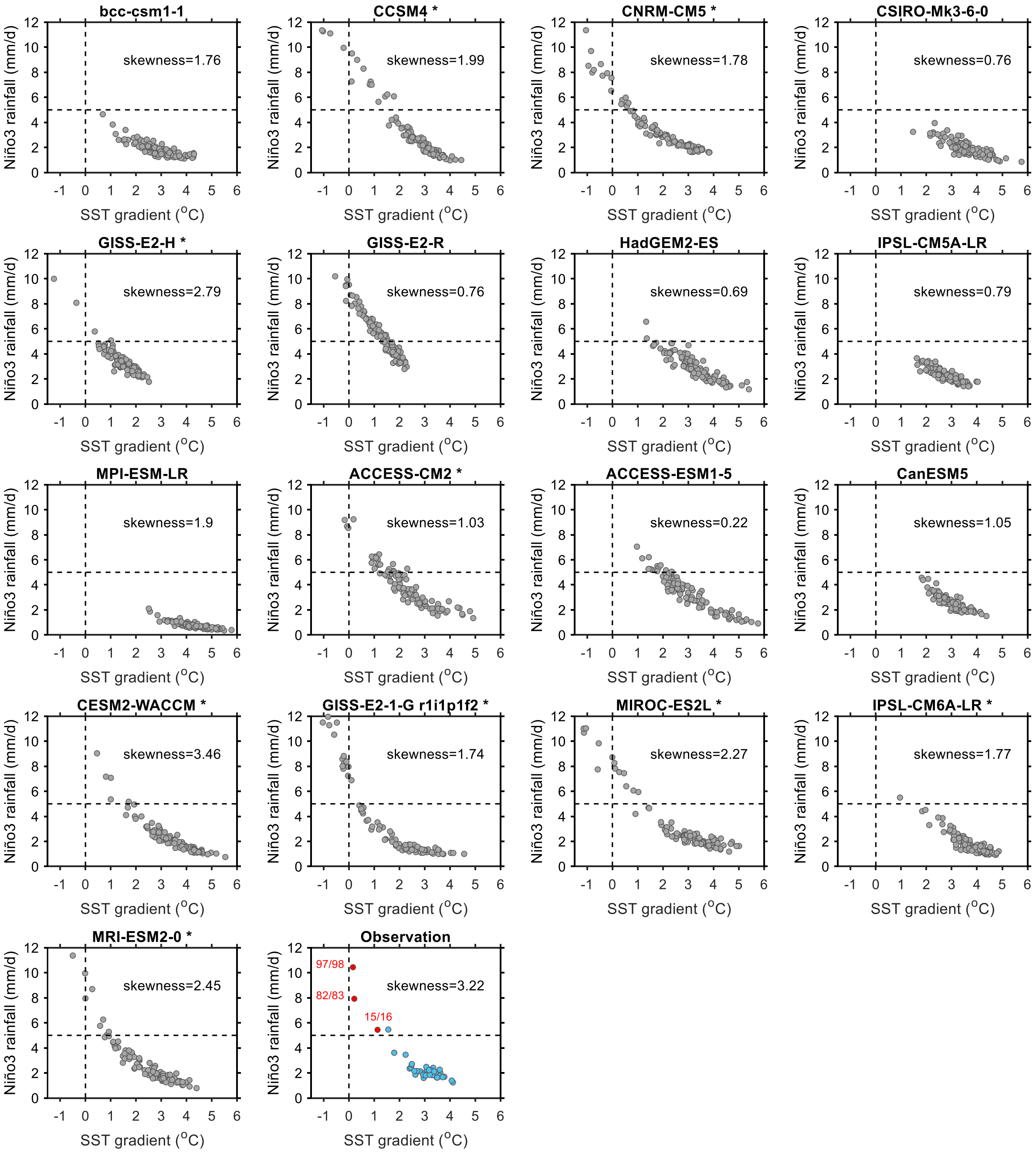

Supplementary Fig. 2 | Assessment of model performance. Relationship between DJF Niño3 (5°S-5°N, 150°W-90°W) rainfall and the SST gradient, calculated as the SST difference between an average of west (2.5^{o}S-2.5^{o}N, 120°E-180°), north (5°N-10°N, 150°W-90°W) and south (5°S-10°S, 160°E-140°W) equatorial Pacific with that in the east (2.5^{o}S-2.5^{o}N, 150°W-90°W) equatorial Pacific, in the 20th century (1900-1999) for each individual model under historical forcings. Also shown is observed relationship in 1979-2023 (Ref.[55,56] in the main text). 9 out of 17 models are

selected (marked by *) according to their ability to (1) simulate the nonlinear Bjerknes feedback with DJF Niño3 rainfall skewness greater than 1.0, and (2) to generate at least one DJF season with Niño3 rainfall greater than 5.0 mm per day (dashed horizonal line)[6].

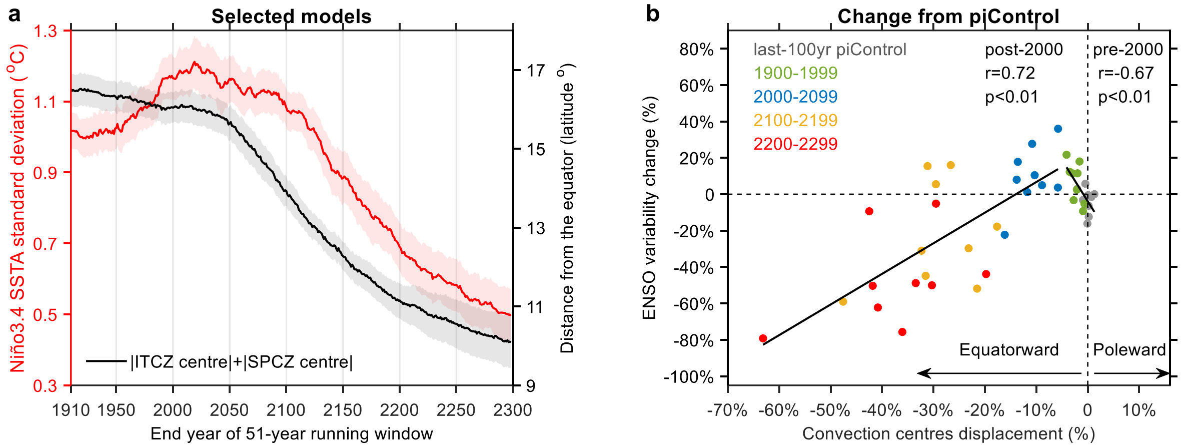

Supplementary Fig. 3 | Nonlinear ENSO response to equatorward convection movement in selected models. a, 51-year running standard deviation of DJF Niño3.4 SST anomaly (red) and the proximity of ITCZ and SPCZ to the equator over ONDJF (black) in the nine selected models under RCP85/SSP585. Years on the x-axis denote the end year of the running window. Solid lines and shadings indicate multi-model mean and 1.0 standard deviation of a total of 10,000 inter-realizations based on a Bootstrap method, respectively. **b,** As in Fig. 2c, d, but for inter-model relationship in the nine selected models. Results based on the nine selected models reinforce our findings.

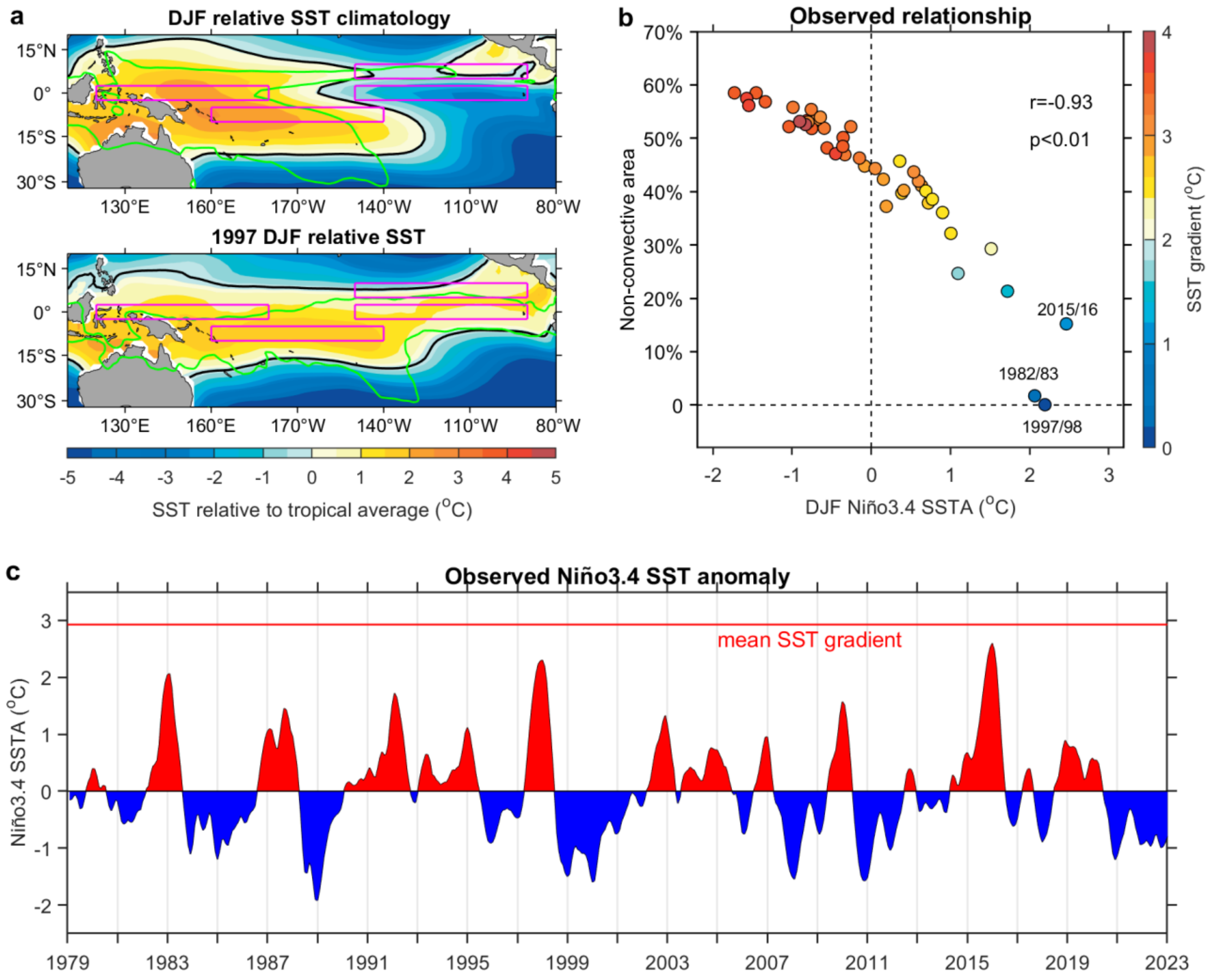

Supplementary Fig. 4 | Defining non-convective area and potential El Niño intensity in observation. A, DJF climatology of relative SST in 1979-2023 (top) and the 1997 extreme El Niño condition (bottom). The relative SST is calculated as SSTs with reference to the tropical (20°S-20°N) average. Black and green contours denote zero line of relative SST and 5 mm per day isoline of rainfall, respectively. Magenta boxes indicate the regions used to calculate the SST gradient. **b,** Relationship between DJF Niño3.4 SST anomaly and non-convective area of the equatorial Pacific areal coverage in percentage in 1979-2023, with color representing magnitude of the SST gradient. **b,** Time series of three-monthly running mean Niño3.4 SST anomaly (bars). Red line denotes the mean DJF SST gradient in the 1979-2023 period. Warm SST anomalies during a strong El Niño event, as in 1997/98, erase much of the climatological west-minus-east and meridional SST gradients in the equatorial Pacific, such that the western Pacific and the off-

equatorial convergence zones move toward the eastern equatorial Pacific, diminishing the non-convective area. Therefore, the zonal and meridional SST gradients set the potential intensity of El Niño.

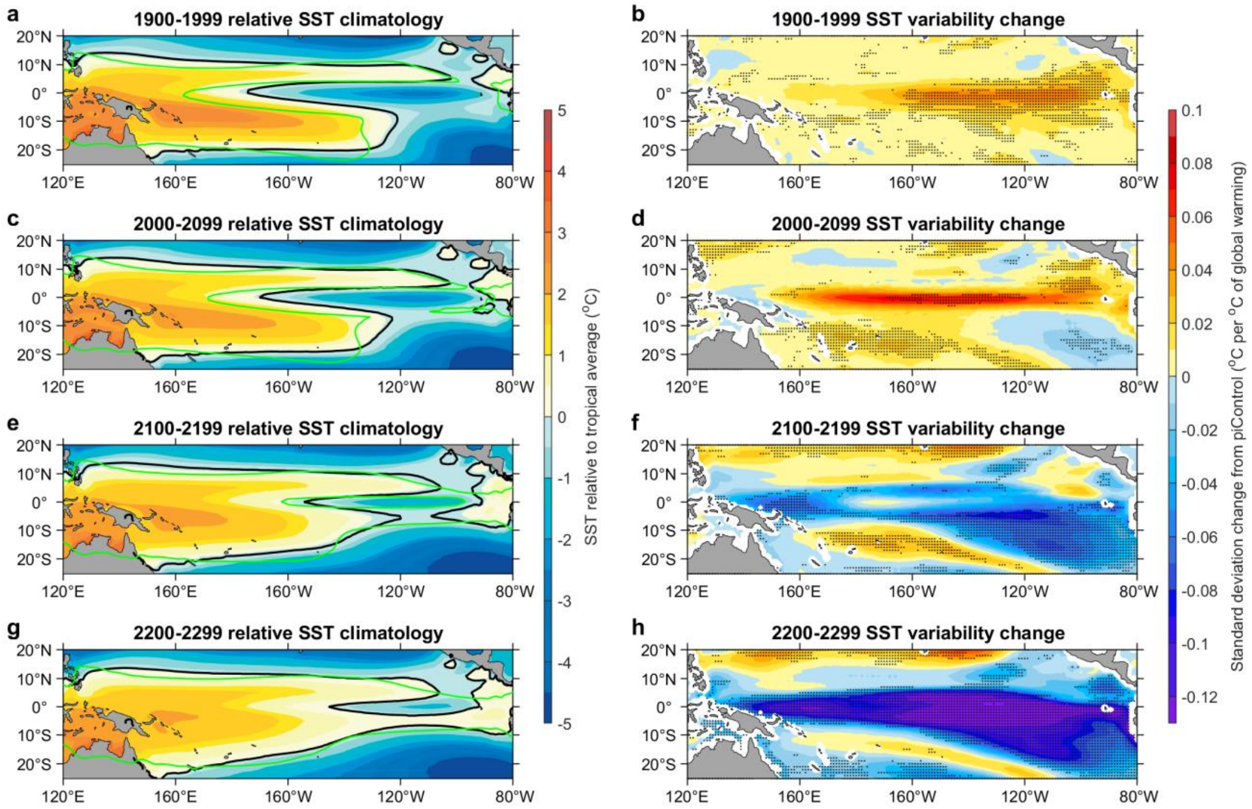

Supplementary Fig. 5 | Shrinking non-convective area and non-unidirectional ENSO variability change. a, Multi-model averaged DJF climatology of relative SST in 1900-1999. **Black** and green contours denote zero line of relative SST and 5 mm per day isoline of rainfall, respectively. **b,** Multi-model averaged SST variability change in 1900-1999 from piControl. Changes are scaled by the increase in global mean surface temperature (global warming) from 1900-1999 and 2000-2099 to facilitate inter-model comparison. **c-h,** As in **a**, **b**, respectively, but for (**c**, **d**) 2000-2099, (**e**, **f**) 2100-2199, and (**g**, **h**) 2200-2299 under RCP85/SSP585. The dotted area indicates regions where more than 75% of models show the same-signed changes. Despite a continuous shrinkage of non-convective area in the equatorial Pacific, ENSO SST variability increases over the 20th and 21st century and decreases after 2100.

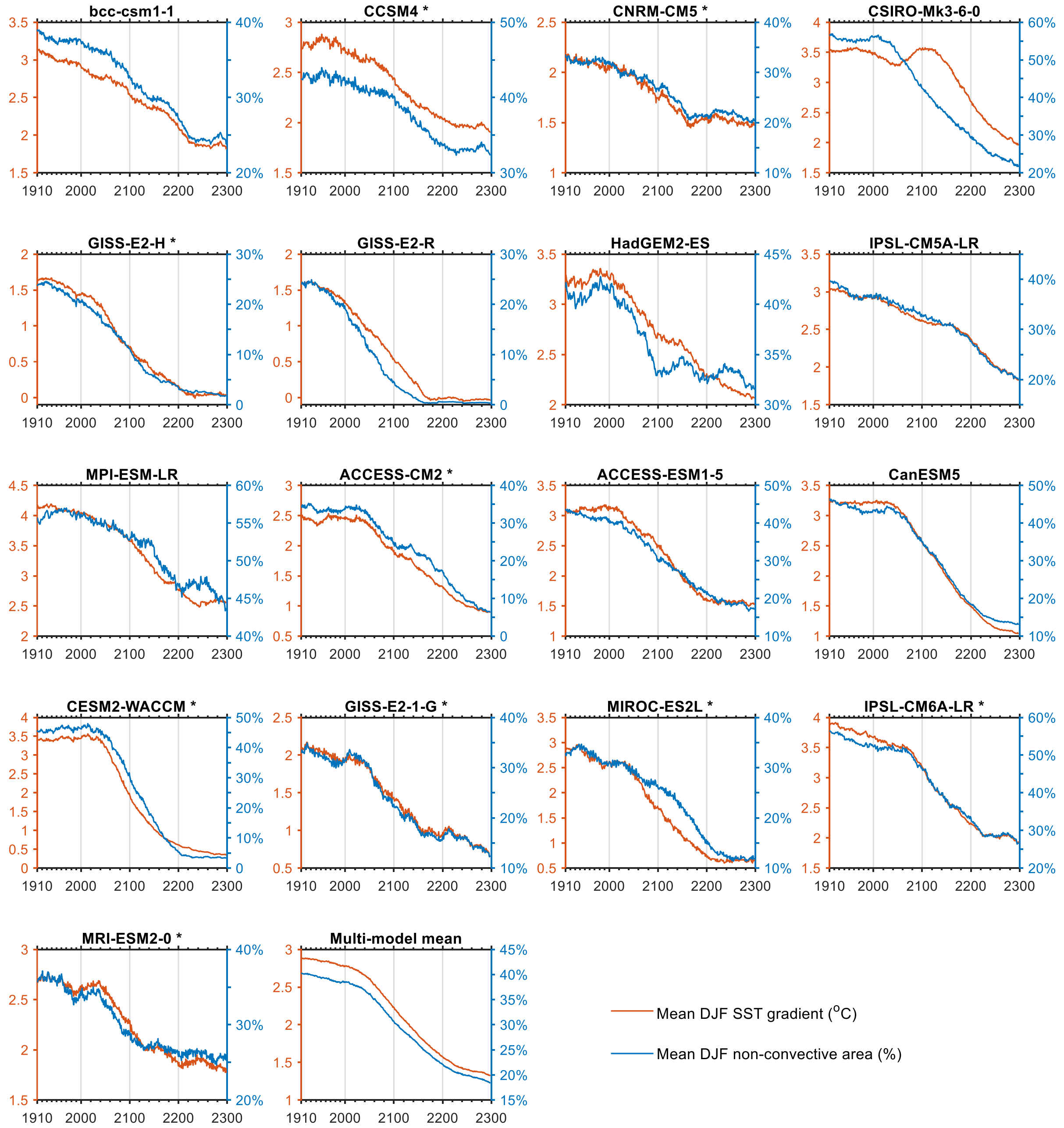

Supplementary Fig. 6 | Coherent decrease of non-convective area and potential El Niño intensity in individual models. Shown are 51-year running means of DJF SST gradient (brown) and non-convective area (blue) for each individual model under RCP85/SSP585. Years on the x-axis denote the end year of the running window. The non-convective area and potential El Niño intensity decrease in tandem under persistent greenhouse warming.

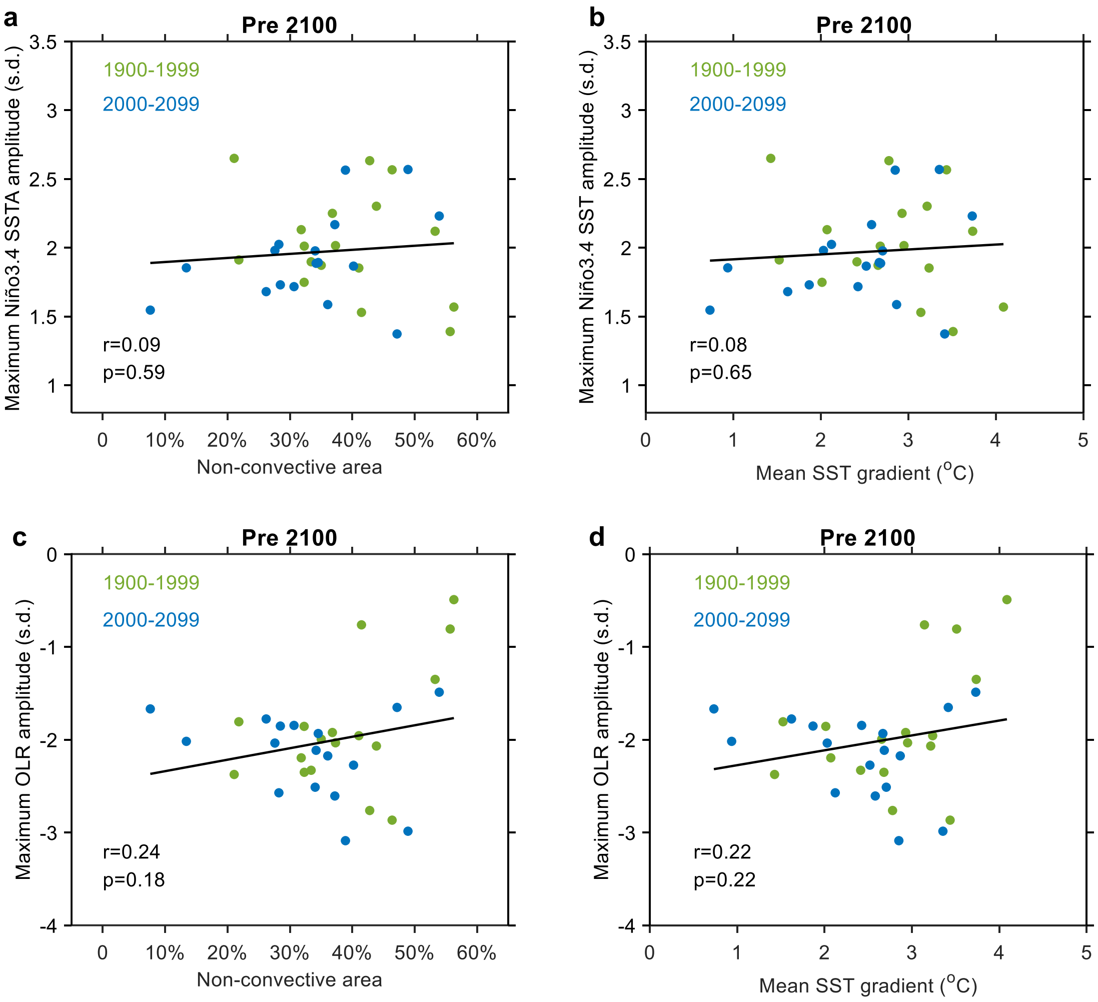

Supplementary Fig. 7 | Little curtailing effect of non-convective area on El Niño intensity before 2100. a, b, As in Fig. 3c, d, respectively, but for the 20th (green dots) and 21st (blue dots) century. **c, d**, As in **a**, **b**, respectively, but for OLR amplitude. Prior to 2100, impact of non-convective area on amplitude of El Niño SST and convective anomalies is not systematic.

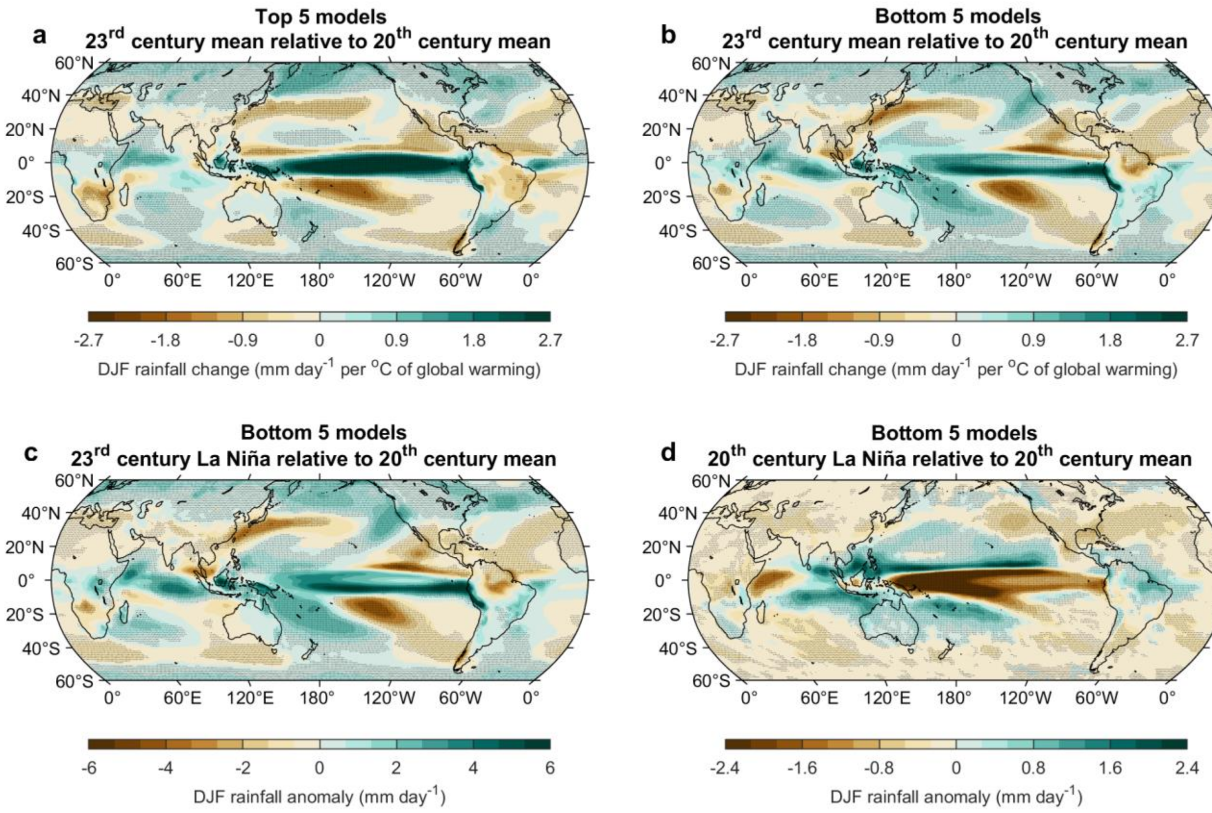

Supplementary Fig. 8 | Mean-state and La Niña-related rainfall changes in models. a, b, Multi-model mean DJF rainfall change in 2200-2299 relative to 1900-1999 DJF climatology in (**a**) top 5 models simulating the largest reductions and (**b**) bottom 5 models simulating the smallest reductions in potential El Niño intensity. Changes are scaled by the increase in global mean surface temperature from 1900-1999 and 2000-2099 to facilitate inter-model comparison. **c, d,** The same as Fig.5 c, de, respectively, but for the bottom 5 models. The dotted area indicates regions where the change or anomaly is statistically significant above the 95% confidence level according to a Bootstrap test. La Niña-induced rainfall in 2200-2299 is dominated by background permanent El Niño-like changes.

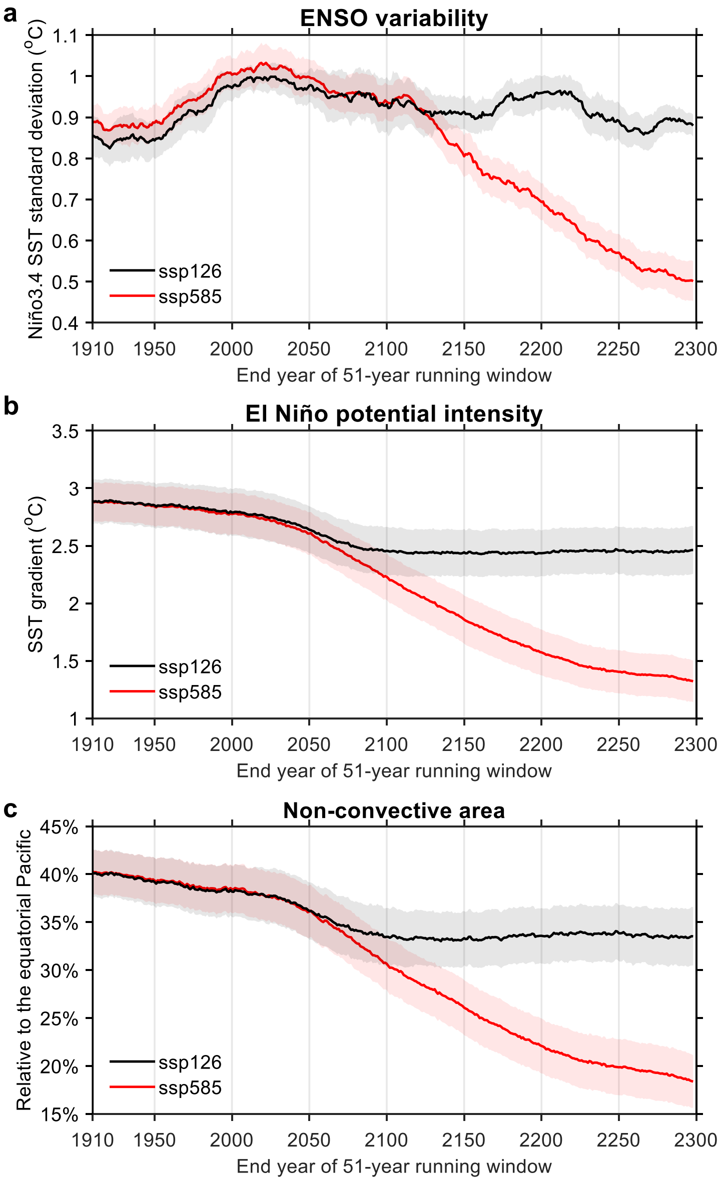

Supplementary Fig. 9 | Disappearance of a permanent El Niño condition under SSP126. a, 51-year running standard deviation of DJF Niño3.4 SST anomaly under **SSP126 (black)** and SSP585 scenarios (red). Years on the x-axis denote the end year of the running window. Solid lines and shadings indicate multi-model mean and 1.0 standard deviation of a total of 10,000 inter-realizations based on a Bootstrap method, respectively. **b, c**, As in **a**, respectively, but for 51-year running means of DJF SST gradient and non-convective area. There is not a persistent decrease in ENSO variability, the potential El Niño intensity or the non-convective area toward 2300 under SSP126, in a sharp contrast to the evolution under SSP585.

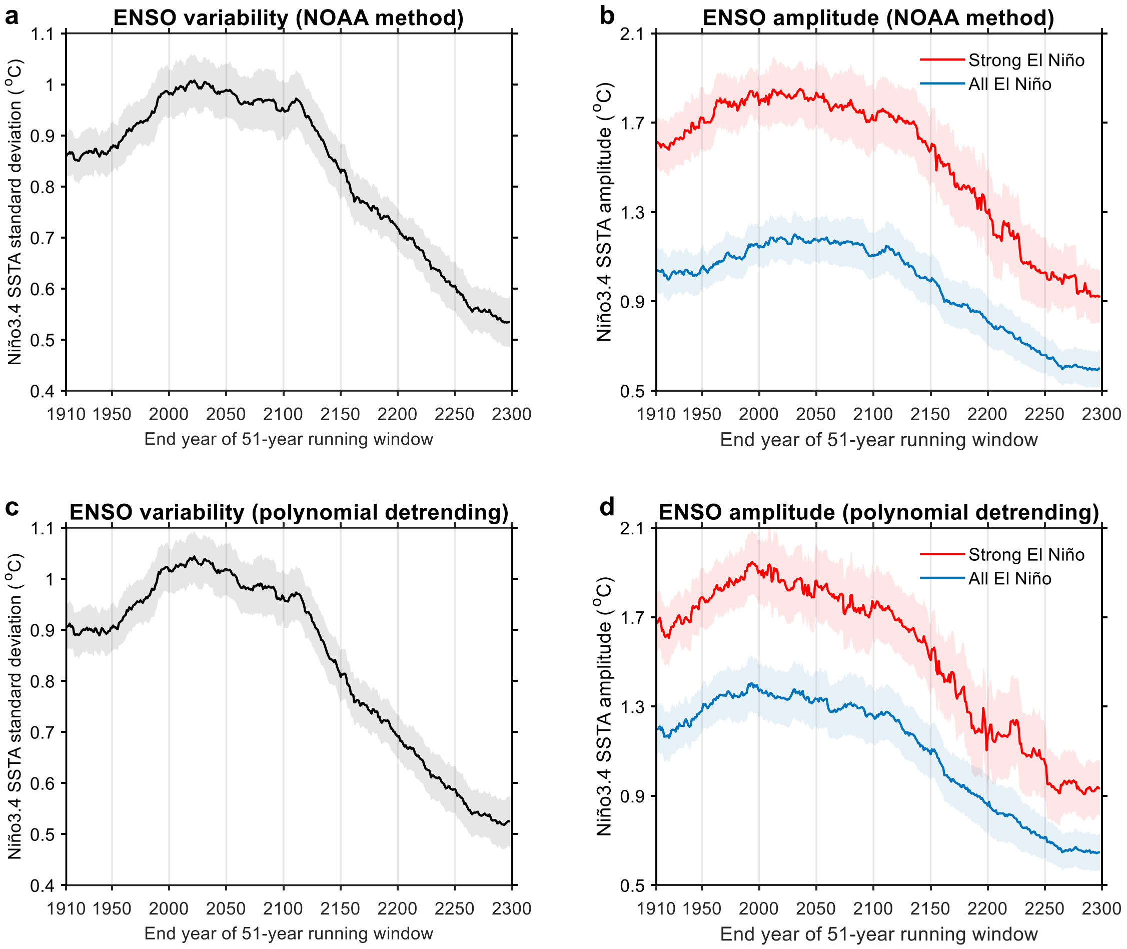

Supplementary Fig. 10 | Sensitivity of ENSO variability change to anomaly definition. **a**, **b**, As in Fig.1c, d, respectively, but for (**a**) 51-year running standard deviation of Niño3.4 SST anomaly (black) and (**b**) 51-year running mean amplitude of Niño3.4 SST anomaly for strong (red) and all (blue) El Niño events using NOAA's method. Specifically, SST anomalies are calculated based on centered 30-year base periods and updated every 5 years, and an El Niño event is defined as when the three-month running mean ONDJF Niño3.4 index exceeds a value of 0.5 standard deviation calculated from the corresponding running periods. **c**, **d**, As in Fig.1c, d, respectively, but using a fifth-order polynomial fit to detrend SST anomalies. The nonlinear ENSO response, with a mild pre-2100 increase followed by a substantial post-2100 decrease, is insensitive to anomaly definitions.

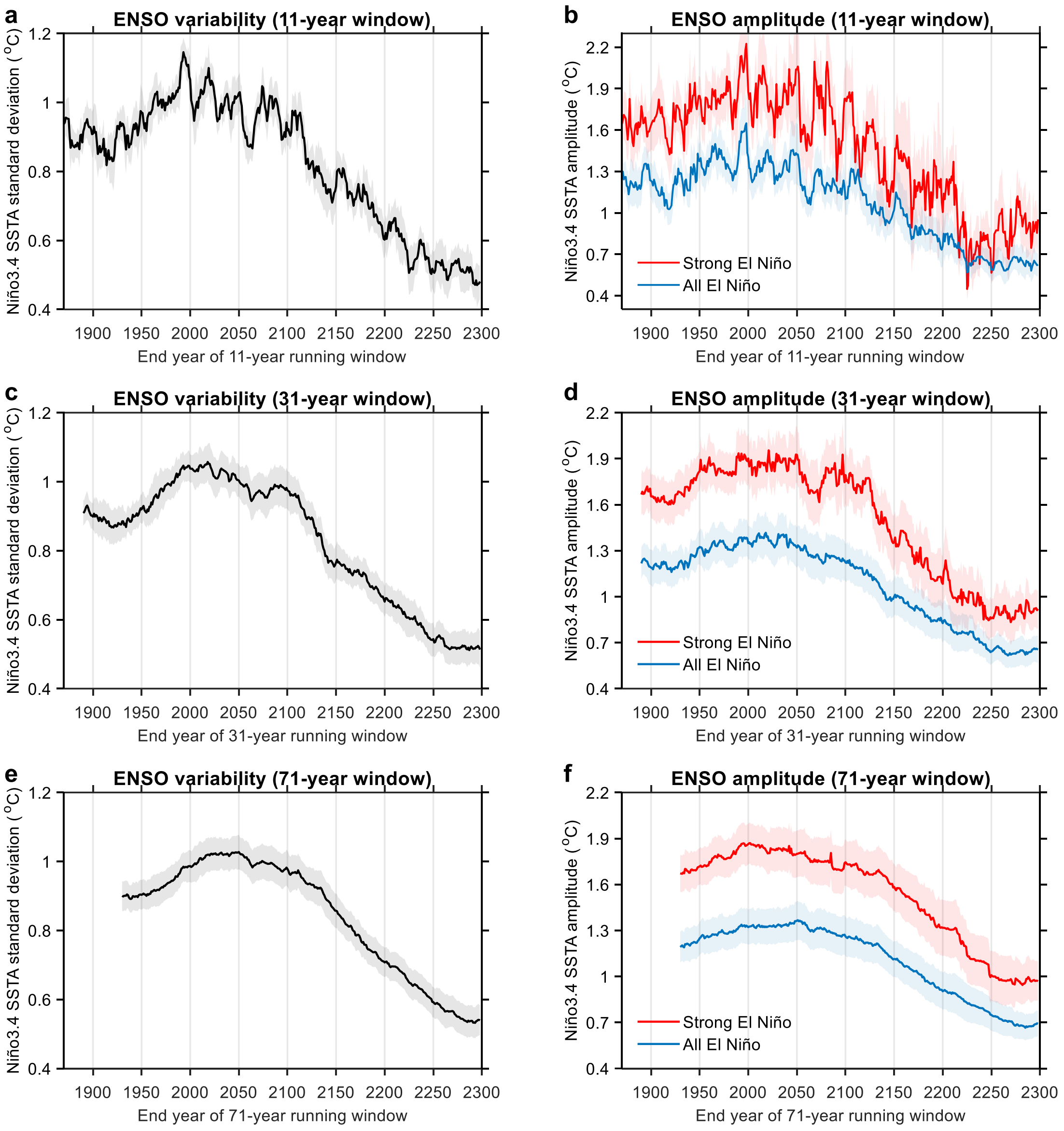

Supplementary Fig. 11 | Sensitivity of ENSO variability change to length of running window. **a**, **b**, As in Fig.1c, d, respectively, but for (**a, b**) 11-year, (**c, d**) 31-year and (**e, f**) 71-year running windowed results, in which SST anomaly is constructed with reference to a corresponding length of running windowed DJF climatology. The nonlinear ENSO response, with a mild pre-2100 increase followed by a substantial post-2100 decrease, is insensitive to length of running windows.

Supplementary Table 1 | Details of models. The nine CMIP5 and eight CMIP6 models used in this study that ran experiments under different warming scenarios (in addition to piControl and historical) till 2300. Where there are multiple realizations, we use the first experiment that is available. Changes in ENSO (Niño3.4) SST variability and potential intensity from piControl to 2200-2299 under RCP85/SSP585 are also shown. **Top five models** that simulate the largest reductions and bottom five models simulating the smallest reductions in potential intensity are **highlighted in bold** and marked with star (*), respectively. Note that not all models have experiments for SSP585 (RCP85) and SSP126 (RCP26).

CMIP5 Model	Institute, Country	Ensemble	Experiment	Potential intensity change (ºC)	ENSO variability change (%)
bcc-csm1-1	BCC, China	r1i1p1	piControl, Historical, RCP26, RCP85	-1.21	-52.84%
CCSM4*	NCAR, USA	r1i1p1	piControl, Historical, RCP26, RCP85	-0.89	-62.24%
CNRM-CM5*	CNRM, France	r1i1p1	piControl, Historical, RCP85	-0.77	-43.87%
CSIRO-Mk3-6-0	CSIRO, Australia	r1i1p1	piControl, Historical, RCP85	-1.41	29.06%
GISS-E2-H	NASA/GISS, USA	r1i1p1	piControl, Historical, RCP26, RCP85	-1.67	-50.02%
**GISS-E2-R**	NASA/GISS, USA	r1i1p1	piControl, Historical, RCP26, RCP85	-1.68	-79.90%
HadGEM2-ES*	MOHC, UK	r1i1p1	piControl, Historical, RCP26, RCP85	-1.09	-31.81%
IPSL-CM5A-LR*	IPSL, France	r1i1p1	piControl, Historical, RCP26, RCP85	-1.06	-27.60%
MPI-ESM-LR	MPI-M, Germany	r1i1p1	piControl, Historical, RCP26, RCP85	-1.60	37.08%

**CMIP6 Model**	**Institute, Country**	**Ensemble**	**Experiment**	**Potential intensity change (ºC)**	**ENSO variability change (%)**
ACCESS-CM2 (Ref.[1,2,3,4])	CSIRO, Australia	r1i1p1f1	piControl, Historical, SSP126, SSP585	-1.44	-75.68%
ACCESS-ESM1-5 (Ref.[5,6,7,8])	CSIRO, Australia	r1i1p1f1	piControl, Historical, SSP126, SSP585	-1.51	-29.70%
**CanESM5** (Ref.[9,10,11,12])	CCCMA, Canada	r1i1p1f1	piControl, Historical, SSP126, SSP585	-2.26	-71.79%
**CESM2-WACCM** (Ref.[13,14,15,16])	NCAR, USA	r1i1p1f1	piControl, Historical, SSP126, SSP585	-3.04	-79.21%
GISS-E2-1-G (Ref.[17,18,19,20])	NASA/GISS, USA	r1i1p1f2	piControl, Historical, SSP126, SSP585	-1.21	-50.31%
**MIROC-ES2L** (Ref.[21,22,23])	JAMSTEC, Japan	r1i1p1f2	piControl, Historical, SSP585	-2.49	-9.37%
**IPSL-CM6A-LR** (Ref.[24,25,26,27])	IPSL, France	r1i1p1f1	piControl, Historical, SSP126, SSP585	-1.91	-48.82%
MRI-ESM2-0* (Ref.[28,29,30,31])	MRI, Japan	r1i1p1f1	piControl, Historical, SSP126, SSP585	-0.86	-5.16%

Supplementary references

1. Dix, M. et al. *CSIRO-ARCCSS ACCESS-CM2 model output prepared for CMIP6 CMIP piControl.* Version 2020201 (Earth System Grid Federation, 2019); https://doi.org/10.22033/ESGF/CMIP6.4311

2.Dix, M. et al. *CSIRO-ARCCSS ACCESS-CM2 model output prepared for CMIP6 CMIP Historical.* Version 2020201 (Earth System Grid Federation, 2019); https://doi.org/10.22033/ESGF/CMIP6.4271

3. Dix, M. et al. *CSIRO-ARCCSS ACCESS-CM2 model output prepared for CMIP6 ScenarioMIP ssp126.* Version 20210201 (Earth System Grid Federation, 2019); https://doi.org/10.22033/ESGF/CMIP6.4319

4. Dix, M. et al. *CSIRO-ARCCSS ACCESS-CM2 model output prepared for CMIP6 ScenarioMIP ssp585.* Version 20210201 (Earth System Grid Federation, 2019); https://doi.org/10.22033/ESGF/CMIP6.4332

5. Ziehn, T. et al. *CSIRO ACCESS-ESM1.5 Model Output Prepared for CMIP6 CMIP piControl.* Version20210201 (Earth System Grid Federation, 2019); https://doi.org/10.22033/ESGF/CMIP6.4312

6. Ziehn, T. et al. *CSIRO ACCESS-ESM1.5 Model Output Prepared for CMIP6 CMIP Historical.* Version20210201 (Earth System Grid Federation, 2019); https://doi.org/10.22033/ESGF/CMIP6.4272

7. Ziehn, T. et al. *CSIRO ACCESS-ESM1.5 Model Output Prepared for CMIP6 ScenarioMIP ssp126.* Version 20210201 (Earth System Grid Federation, 2019); https://doi.org/10.22033/ESGF/CMIP6.4320

8. Ziehn, T. et al. *CSIRO ACCESS-ESM1.5 Model Output Prepared for CMIP6 ScenarioMIP ssp585.* Version 20210201 (Earth System Grid Federation, 2019); https://doi.org/10.22033/ESGF/CMIP6.4333

9. Swart, N. C. et al. *CCCma CanESM5 Model Output Prepared for CMIP6 CMIP piControl.* Version 20210201 (Earth System Grid Federation, 2019); https://doi.org/10.22033/ESGF/CMIP6.3673

10. Swart, N. C. et al. *CCCma CanESM5 Model Output Prepared for CMIP6 CMIP Historical.* Version 20210201 (Earth System Grid Federation, 2019); https://doi.org/10.22033/ESGF/CMIP6.3610

11. Swart, N. C. et al. *CCCma CanESM5 Model Output Prepared for CMIP6 ScenarioMIP ssp126.* Version 20210201 (Earth System Grid Federation, 2019); https://doi.org/10.22033/ESGF/CMIP6.3683
12. Swart, N. C. et al. *CCCma CanESM5 Model Output Prepared for CMIP6 ScenarioMIP ssp585.* Version 20210201 (Earth System Grid Federation, 2019); https://doi.org/10.22033/ESGF/CMIP6.3696
13. Danabasoglu, G. *NCAR CESM2-WACCM Model Output Prepared for CMIP6 CMIP piControl.* Version 20210201 (Earth System Grid Federation, 2019); https://doi.org/10.22033/ESGF/CMIP6.10094
14. Danabasoglu, G. *NCAR CESM2-WACCM Model Output Prepared for CMIP6 CMIP Historical.* Version 20210201 (Earth System Grid Federation, 2019); https://doi.org/10.22033/ESGF/CMIP6.10071
15. Danabasoglu, G. *NCAR CESM2-WACCM Model Output Prepared for CMIP6 ScenarioMIP ssp126.* Version 20210201 (Earth System Grid Federation, 2019); https://doi.org/10.22033/ESGF/CMIP6.10100
16. Danabasoglu, G. *NCAR CESM2-WACCM Model Output Prepared for CMIP6 ScenarioMIP ssp585.* Version 20210201 (Earth System Grid Federation, 2019); https://doi.org/10.22033/ESGF/CMIP6.10115
17. NASA Goddard Institute For Space Studies (NASA/GISS). *NASA-GISS GISS-E2.1G Model Output Prepared for CMIP6 CMIP piControl.* Version 20210201 (Earth System Grid Federation, 2018); https://doi.org/10.22033/ESGF/CMIP6.7380
18. NASA Goddard Institute For Space Studies (NASA/GISS). *NASA-GISS GISS-E2.1G Model Output Prepared for CMIP6 CMIP Historical.* Version 20210201 (Earth System Grid Federation, 2018); https://doi.org/10.22033/ESGF/CMIP6.7127
19. NASA Goddard Institute For Space Studies (NASA/GISS). *NASA-GISS GISS-E2.1G Model Output Prepared for CMIP6 ScenarioMIP ssp126* Version 20210201 (Earth System Grid Federation, 2020); https://doi.org/10.22033/ESGF/CMIP6.7410
20. NASA Goddard Institute For Space Studies (NASA/GISS). *NASA-GISS GISS-E2.1G Model Output Prepared for CMIP6 ScenarioMIP ssp585* Version 20210201 (Earth System Grid Federation, 2020); https://doi.org/10.22033/ESGF/CMIP6.7460

21. Hajima, T. et al. *MIROC MIROC-ES2L Model Output Prepared for CMIP6 CMIP piControl.* Version 20210201 (Earth System Grid Federation, 2019); https://doi.org/10.22033/ESGF/CMIP6.5710

22. Hajima, T. et al. *MIROC MIROC-ES2L Model Output Prepared for CMIP6 CMIP Historical.* Version 20210201 (Earth System Grid Federation, 2019); https://doi.org/10.22033/ESGF/CMIP6.5602

23. Tachiiri K. et al. *MIROC MIROC-ES2L Model Output Prepared for CMIP6 ScenarioMIP ssp585.* Version 20210201 (Earth System Grid Federation, 2019); https://doi.org/10.22033/ESGF/CMIP6.5770

24. Boucher, O. et al. *IPSL IPSL-CM6A-LR Model Output Prepared for CMIP6 CMIP piControl.* Version 20210201 (Earth System Grid Federation, 2019); https://doi.org/10.22033/ESGF/CMIP6.5251

25. Boucher, O. et al. *IPSL IPSL-CM6A-LR Model Output Prepared for CMIP6 CMIP Historical.* Version 20210201 (Earth System Grid Federation, 2019); https://doi.org/10.22033/ESGF/CMIP6.5195

26. Boucher, O. et al. *IPSL IPSL-CM6A-LR Model Output Prepared for CMIP6 ScenarioMIP ssp126.* Version 20210201 (Earth System Grid Federation, 2019); https://doi.org/10.22033/ESGF/CMIP6.5262

27. Boucher, O. et al. *IPSL IPSL-CM6A-LR Model Output Prepared for CMIP6 ScenarioMIP ssp585.* Version 20210201 (Earth System Grid Federation, 2019); https://doi.org/10.22033/ESGF/CMIP6.5271

28. Yukimoto, S. et al. *MRI MRI-ESM2.0 Model Output Prepared for CMIP6 CMIP piControl.* Version 20210201 (Earth System Grid Federation, 2019); https://doi.org/10.22033/ESGF/CMIP6.6900

29. Yukimoto, S. et al. *MRI MRI-ESM2.0 Model Output Prepared for CMIP6 CMIP Historical.* Version 20210201 (Earth System Grid Federation, 2019); https://doi.org/10.22033/ESGF/CMIP6.6842

30. Yukimoto, S. et al. *MRI MRI-ESM2.0 Model Output Prepared for CMIP6 ScenarioMIP ssp126.* Version 20210201 (Earth System Grid Federation, 2019); https://doi.org/10.22033/ESGF/CMIP6.6909

31. Yukimoto, S. et al. *MRI MRI-ESM2.0 Model Output Prepared for CMIP6 ScenarioMIP ssp585*. Version 20210201 (Earth System Grid Federation, 2019); https://doi.org/10.22033/ESGF/CMIP6.6929
